# Exploring the role of lipids in intercellular conduits: breakthroughs in the pipeline

**DOI:** 10.3389/fpls.2013.00504

**Published:** 2013-12-10

**Authors:** Elise Delage, Chiara Zurzolo

**Affiliations:** Unité de Trafic Membranaire et Pathogenèse, Département de Biologie Cellulaire et Infection, Institut PasteurParis, France

**Keywords:** intercellular communication, plasmodesmata, tunneling nanotubes, membrane lipids, phosphoinositides, imaging techniques

## Abstract

It has been known for more than a century that most of the plant cells are connected to their neighbors through membranous pores perforating the cell wall, namely plasmodesmata (PDs). The recent discovery of tunneling nanotubes (TNTs), thin membrane bridges established between distant mammalian cells, suggests that intercellular communication mediated through cytoplasmic continuity could be a conserved feature of eukaryotic organisms. Although TNTs differ from PDs in their formation and architecture, both are characterized by a continuity of the plasma membrane between two cells, delimiting a nanotubular channel supported by actin-based cytoskeleton. Due to this unusual membrane organization, lipids are likely to play critical roles in the formation and stability of intercellular conduits like TNTs and PDs, but also in regulating the transfer through these structures. While it is crucial for a better understanding of those fascinating communication highways, the study of TNT lipid composition and dynamics turned out to be extremely challenging. The present review aims to give an overview of the recent findings in this context. We will also discuss some of the promising imaging approaches, which might be the key for future breakthroughs in the field and could also benefit the research on PDs.

## INTRODUCTION

**FIGURE 1:**
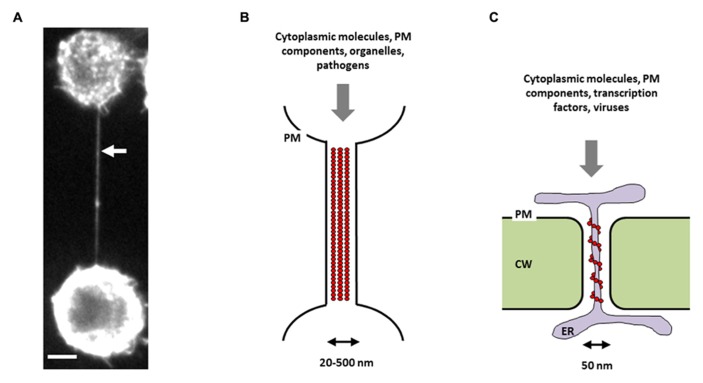
**Intercellular conduits in mammalian and plant cells**. **(A)** Picture of a TNT connecting two neuronal CAD cells in culture. Cells were stained with wheat germ agglutinin in order to visualize TNT membrane, then fixed, and imaged by spinning-disk fluorescence microscopy. White arrow indicates a TNT connecting two remote cells. Scale bar = 5 μM. Schematic representations of a TNT **(B)** and a PD **(C)**. PM = plasma membrane, CW = cell wall, ER = endoplasmic reticulum, red circles = actin-based cytoskeleton. Note the absence of a midbody, which excludes the possibility that this structure could be an intercellular bridge.

The existence of “cytoplasmic bridges” between plant cells was first reported by Tangl (1880). These structures, later named “plasmodesmata” (PDs), are thin plasma-membrane lined pores embedded in the cell wall and allowing direct cell-to-cell transmission of materials and signals (Kragler, 2013). More recently, the discovery that many different mammalian cell types can also be connected by cytoplasmic bridges, namely tunneling nanotubes (TNTs; **Figure 1A**), suggests that this type of communication is not a hallmark of plant cells (Rustom et al., 2004; Rustom, 2009). Like PDs, TNTs are thin membranous channels supported by the actin cytoskeleton mediating intercellular communication through cytoplasmic continuity (**Figures  1B**,**C**). These structures are dynamic and heterogenous and contrary to other types of membrane protrusions, such as filopodia, do not touch the substrate in cell culture (Rustom et al., 2004; Abounit and Zurzolo, 2012). Although the lack of known molecular markers hampers the identification of TNTs within tissues, several recent studies described the presence of TNT-like structures *in vivo* (Chinnery et al., 2008; Pyrgaki et al., 2010; Lou et al., 2012; Seyed-Razavi et al., 2013). If TNT diameter (20–500 nm) is comparable to PD diameter (~50 nm), TNT length is highly variable and can extend up to several cell diameters (~100 μm), whereas the length of PD is determined by the cell wall thickness (Gerdes et al., 2007). Another difference between the two structures is that TNTs lack the central desmotubule (membranous rod of appressed endoplasmic reticulum), which is typical of most PDs (**Figures 1B**,**C**).

In addition, while primary PDs result from incomplete cell plate formation during cytokinesis, TNTs, like secondary PDs, are formed *de novo* and can be observed between heterotypic cells (Gerdes et al., 2007). Therefore TNTs are very dynamic structures which can be formed after cells previously in contact detach from one another, or can arise from the extension of filopodia-like protrusions toward neighboring cells (Abounit and Zurzolo, 2012; Kimura et al., 2012). Although some early steps in TNT genesis have been highlighted, the molecular pathways involved in their formation are still unclear (Marzo et al., 2012; Gousset et al., 2013). In addition, the structural (e.g., length/diameter, presence of microtubules, open-endedness) and functional (e.g., type of transferred cargoes/signals) diversity observed among TNT-like structures in various cell-types suggests that they may also differ in their formation mechanisms (Abounit and Zurzolo, 2012). A wide variety of cellular materials, such as cytoplasmic molecules, plasma membrane (PM) components, vesicles derived from various organelles, and even whole organelles (e.g., mitochondria) have been shown to transfer through TNTs (Marzo et al., 2012; Gerdes et al., 2013). Furthermore, TNTs can be “hijacked” by different pathogens, such as bacteria, viruses, or prions, and might represent a general way for pathogen spreading (Hurtig et al., 2010; Marzo et al., 2012). Therefore these structures attracted much attention in cell biology over the last decade. While some TNT constituents, such as actin and myosin which are also found in PDs, have been identified (Abounit and Zurzolo, 2012), the lipid composition of their membrane remains largely unknown. Nevertheless, this question is of major interest because the peculiar conformation of intercellular conduits like TNTs and PDs suggests that lipids play crucial roles in their establishment and function. Indeed, although lipids have for a long time been considered as passive building blocks of cellular membranes, their active role in many cellular processes such as membrane trafficking, cytoskeleton remodeling and signaling, is now widely recognized (Takenawa and Itoh, 2001; Wenk, 2005). Specifically, some membrane lipids, such as phosphoinositides or sphingolipids, can be precursors of signaling molecules and can also directly interact with proteins, thus regulating their activity or subcellular location (Wenk, 2005; Delage et al., 2013). In addition, lipids can segregate in membrane nano and microdomains such as rafts, “small (10–200 nm), heterogeneous, highly dynamic, sterol- and sphingolipid-enriched domains that compartmentalize cellular processes” (Pike, 2006; Simons and Sampaio, 2011) involved in many biological events (Sonnino and Prinetti, 2013).

The present review aims to emphasize the multiple different functions that lipids might exert in TNTs and to summarize the current knowledge on this topic. In addition, we will discuss some of the promising imaging techniques that might be crucial to decipher lipid organization within nanotubular intercellular conduits such as TNTs or PDs.

### MEMBRANE LIPIDS AS POSSIBLE KEYSTONES OF TNT STRUCTURE AND FUNCTION: THE PREMISES

**FIGURE 2:**
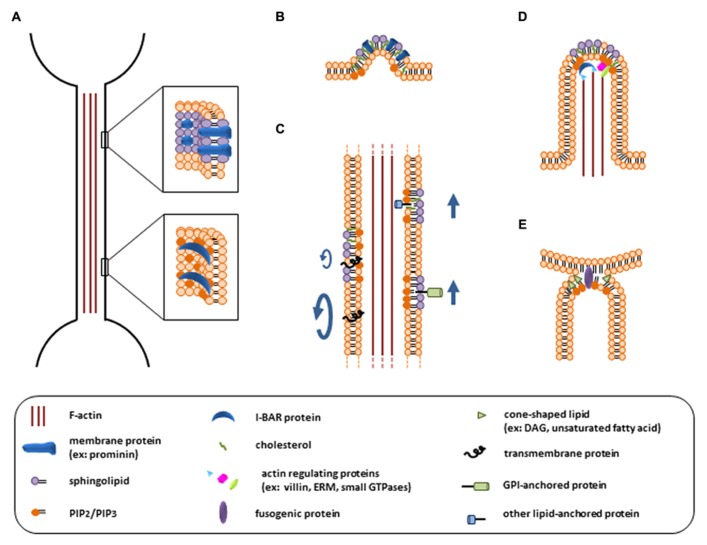
**Potential key roles of membrane lipids in TNT formation and function**. **(A)** Stabilization of the highly curved TNT membrane by lipid and protein nanodomains such as rafts, or by I-BAR proteins recruited via phosphoinositide binding. **(B)** Induction of membrane tubular budding by lipid and protein clustering. **(C)** Influence of membrane organization on TNT transfer function. Ordered lipid domains can restrict the lateral diffusion of membrane proteins (*left*) or promote their targeting and subsequent transfer through TNTs (*right*). **(D)** Regulation of actin cytoskeleton by phosphoinositides during TNT formation via filopodia-like protrusions. **(E)** Facilitation of membrane fusion by lipid physical properties.

It is noteworthy that, similar to PD membrane, the membrane delimiting TNTs is characterized by a strong curvature, which suggests that its lipid composition differs from the surrounding PM (**Figure 2A**). Indeed, different studies using artificial membrane nanotubes and theoretical predictions highlighted the reciprocal influence of membrane curvature and lipid segregation (Callan-Jones et al., 2011; Kabaso et al., 2012). Lipid sorting can reduce the energy cost of membrane bending, which depends on the deformability of the bilayer and on the molecular shape of its lipid components (Callan-Jones et al., 2011; Lokar et al., 2012). Interestingly, several recent papers indicated that the clustering of lipid and protein nanodomains with an affinity for highly curved membrane regions may induce a tubular budding of the membrane, even in absence of a pushing or pulling force from the cytoskeleton (**Figure 2B**; Farsad and Camilli, 2003; Gimsa et al., 2007; Iglic et al., 2007; Römer et al., 2007). The accumulation of specific membrane domains (or rafts) enriched in proteins that preferentially localize to cylindrical membrane protrusions and generate anisotropy, like the membrane protein prominin, could also be crucial for the stability of those structures (**Figure 2A**; Iglič, 2006; Veranič et al., 2008; Hurtig et al., 2010; Kabaso et al., 2012). In addition, an enrichment of ordered lipid domains could influence TNT transfer function by restricting the lateral diffusion of membrane components or by targeting membrane proteins, such as glycosylphosphatidylinositol (GPI)-anchored proteins, to TNTs for intercellular transfer (**Figure 2C**; Veranič etal., 2008; Tilsner et al., 2010). Interestingly, the presence of rafts in filopodia and other membrane protrusions has been highlighted (Corbeil et al., 2001; Huttner and Zimmerberg, 2001; Gupta and DeFranco, 2003). Furthermore several evidences pointing towards a possible enrichment of membrane rafts in PDs have been reported (Tilsner et al., 2010; Cacas et al., 2012) suggesting that this could be a common feature of thin tubular membrane structures.

Lipid constituents of the PM can also influence its curvature via their tight interplay with membrane-bending proteins, such as Bin/amphiphysin/Rvs (BAR) domain-containing proteins (**Figure 2A;** Rao and Haucke, 2011). In contrast to other members of the BAR domain family which generate membrane invaginations, inverse BAR (I-BAR) domains recognize negative curvature and induce membrane protrusions (Saarikangas et al., 2009). I-BAR proteins have been implicated in filopodia generation in different cell types (Millard et al., 2005; Mattila et al., 2007; Saarikangas et al., 2009) and might play a similar role in TNT formation. The I-BAR domain electrostatically interacts with negatively charged phospholipids, with a stronger affinity for phosphatidylinositol 4,5-biphosphate [PI(4,5)P_2_] (**Figure 2A**; Mattila et al., 2007; Saarikangas et al., 2009). Interestingly, I-BAR proteins can also bind many different regulators of the actin cytoskeleton and their main role in filopodia formation may be achieved through the coupling of membrane protrusion and actin filament formation (**Figure 2D**; Ahmed et al., 2010).

The structural and functional relation between the actin cytoskeleton and lipid constituents of the PM is far from being limited to I-BAR proteins. It has been suggested that components of the actin cytoskeleton exert an ordering effect on the lipid bilayer and could contribute to assemble membrane rafts (Chichili and Rodgers, 2009; Gowrishankar et al., 2012). Various raft structural and functional features require an intact actin cytoskeleton. In turn, proteins and lipids involved in cytoskeleton regulation or anchorage to the PM are found associated with rafts (**Figure 2D**). Notably, the inner leaflet of membrane rafts is enriched with phosphoinositides, such as PI(4,5)P_2_ and phosphatidylinositol 3,4,5-trisphosphate (PIP_3_), which emerged as major regulators of cytoskeleton structure and dynamics (Saarikangas et al., 2010). In a nutshell, phosphoinositides positively regulate proteins that promote actin polymerization and inhibit proteins that induce filament disassembly (**Figure 2D**). They also contribute to anchor actin filament to the PM through protein linkers, such as ezrin-radixin-moesin (ERM) proteins, key proteins in the formation of PM protrusions (Chichili and Rodgers, 2009; Saarikangas et al., 2010). Specifically, the role of PI(4,5)P_2_ and PIP_3_ in filopodia formation, in relation with the actin cytoskeleton, has been widely documented (Arjonen et al., 2011; Khurana and George, 2011). Interestingly, sphingosine 1-phosphate can also induce filopodia formation through the activation of ERM proteins (Gandy et al., 2013). Because actin plays a major role in TNT generation via filopodia-like protrusions (Abounit and Zurzolo, 2012; Gousset et al., 2013), we speculate that phosphoinositides and sphingolipids are likely to be involved in this process.

Another crucial step of TNT formation in which lipids may play an important role is the fusion of the two plasmalemmas, leading to cytoplasmic continuity of the connected cells. Indeed, although fusion probably requires fusogenic proteins it is sensitive to membrane lipid composition. Depending on their intrinsic shape and their position in the inner or outer leaflet of the bilayer, lipids may differently affect the propensity of the membranes to merge. For example, lipids with an inverted cone shape, such as phosphoinositides or lysophospholipids, can promote the fusion of membranes when located in the distal leaflet, whereas cone-shaped lipids, like cis-unsaturated fatty acids or diacylglycerol, promote it when located in the proximal leaflet (**Figure 2E**; Chernomordik and Kozlov, 2003; Larijani and Poccia, 2012).

### MEMBRANE LIPIDS AS POSSIBLE KEYSTONES OF TNT STRUCTURE AND FUNCTION: THE CURRENT KNOWLEDGE

In accordance with what has been hypothesized from theoretical studies, experimental data suggested the presence of specific lipid domains in the membrane lining TNTs. Indeed, by using the raft marker ostreolysin (Oly) Iglič and coworkers recently highlighted the presence of cholesterol-sphingomyelin nanodomains within TNTs in a T24 urothelial cancer cell line (Lokar et al., 2012). In contrast to Oly, very little binding of cholera toxin B, which binds to the raft-specific ganglioside GM1, was observed along TNTs, and immunofluorescence studies do not reveal the presence of caveolin-1 and flotillin-1 raft markers (Bickel, 2002). On the other hand, addition of the cholesterol depletion agent methyl-β-cyclodextrin and the growth in cholesterol-free medium were shown to reduce the number of TNTs, suggesting a role for cholesterol in the stability of these structures (Lokar et al., 2012). Cholesterol is expected to be more present in the external leaflet of TNT to minimize the bending energy cost. According to their experimental results and computational model, the authors proposed that cholesterol depletion from the outer leaflet may reduce the area difference between the two leaflets, thus favoring more planar conformation and leading to the detachment of TNT from the parent PM (Lokar et al., 2012).

To our knowledge, no other data regarding the lipid composition of TNTs has been reported in the literature thus far. However, indirect evidences support the role of PIP_3_ in TNT formation. Wang and collaborators (Wang et al., 2011) recently highlighted the involvement of the Akt/phosphatidylinositol 3-kinase (PI3K)/mTor pathway in TNT generation in astrocytes under H_2_O_2_ treatment. Interestingly, they observed a drastic reduction of stress-induced TNT formation in astrocytes treated with PI3K and mTor inhibitors or expressing the Akt dominant negative mutant, whereas expression of the constitutive form of Akt increased the number of connections. They also reported an increase of Akt and PI3K phosphorylated forms upon H_2_O_2_ treatment. In this context, PIP_3_ formation thus appears as an important step for TNT genesis.

In addition, recent data obtained in our laboratory using CAD cells, a mouse neuronal cell line of catecholaminergic origin, also suggested the importance of PIP_3_ for TNT generation (Gousset et al., 2013). It was shown that expression of the unconventional molecular motor myosin 10 (Myo10) increases the number of functional TNTs and the vesicle transfer between connected cells. A point mutation in the second pleckstrin homology of Myo10, which impairs its binding to PIP_3_, hindered the ability of Myo10 to induce TNT formation. Thus, in accordance with what has been reported for filopodia (Plantard et al., 2010; Lu et al., 2011), Myo10 recruitment to the PM through PIP_3_ binding seems to be necessary for Myo10 role in TNT induction. However, contrary to what has been shown for astrocytes, no correlation between Akt activation and TNT formation has been observed in CAD cells, suggesting a PI3K dependent but Akt independent pathway (Gousset et al., 2013).

### PROMISING IMAGING TECHNIQUES FOR FURTHER BREAKTHROUGHS

Although the cues for a critical role of lipids in TNT are considerable, research in the field has been hampered by technical limitations. While PD-enriched fractions can be obtained from plant cell walls, thus allowing biochemical approaches (Maule et al., 2011), isolation of “bona-fide“ TNT from the cell bodies is quite challenging. Indeed, although the isolation and proteomic study of bridging conduits connecting macrophages has been recently reported (Kadiu and Gendelman, 2011), the technique used to harvest those thicker and chemotaxis-driven protrusions does not appear to be applicable to the isolation of canonical TNTs. On the other hand, imaging techniques are to date the most suitable approaches to study TNT components. As a sensitive and versatile technique, and because it allows to image live cells, fluorescence microscopy is particularly well-suited to investigate lipid organization in the membrane of intercellular conduits.

While classical studies consist of imaging the distribution of the fluorescence intensity from a given fluorophore (lipid-binding probes, lipid analogs, or fluorescently tagged proteins used as raft markers; Hullin-Matsuda et al., 2009), several recent techniques made it possible to monitor additional fluorescence parameters such as spectral shifts and anisotropy (Braeckmans et al., 2011; Bastos et al., 2012). Combined with the utilization of probes whose physical properties are influenced by membrane packing order, such as 2-dimethylamino-6-lauroylnaphthalene (Laurdan), these techniques could be valuable to investigate the presence of lipid rafts in intercellular conduits (Dodes Traian et al., 2011; Owen et al., 2011). Because membrane organization can influence probe fluorescence lifetimes, this question could also be addressed by fluorescence lifetime imaging microscopy (FLIM; Bastos et al., 2012). Furthermore FLIM coupled with Förster resonance energy transfer (FRET), FLIM-FRET, can be applied to cellular membranes to address major questions, like the formation of lipid domain clusters, probe partition between specific membrane domains, or interaction of membrane components within a given domain (de Almeida et al., 2009; Stöckl and Herrmann, 2010). This technique has been successfully used by Konig et al. (2008) to demonstrate the association of the cortical actin meshwork with PIP_3_-enriched compartments of the PM. Therefore a similar approach could yield important information on lipid/actin interactions within intercellular conduits.

Because lateral organization of the membrane affects the mobility of its constituents, sub-resolution membrane domains can also be analyzed thanks to fluorescence microscopy techniques based on molecular dynamics assessment (Owen et al., 2009). This include highly sensitive single molecule techniques like fluorescence correlation spectroscopy (FCS) and single particle tracking (SPT), which could allow to address crucial questions regarding membrane domains, such as lipid–lipid and lipid–protein interactions within the tubular membranes, which cannot be unraveled with conventional optical methods (Chiantia et al., 2009; Owen et al., 2009).

A major issue when studying lipid organization in cellular membranes is that the predicted size of lipid nanodomains is below the resolution limit of classical optical microscopes (Pike, 2006; Owen et al., 2012). The development of super-resolution techniques that allow to image structures beyond the diffraction limit, such as photo-activated localization microscopy (PALM), stimulated emission depletion (STED) microscopy, and structured illumination microscopy (SIM), greatly improved the possibilities in the field. Combinations of far-field super-resolution techniques with approaches such as FLIM, FCS, and SPT have substantially increased our perception of lipid organization in biological membranes during the past few years (Owen et al., 2009) and may represent the most promising way to decipher lipid organization and dynamics within TNTs or PDs.

An alternative to fluorescence microscopy for high-resolution study of membrane organization within intercellular conduits might be the utilization of scanning or transmission electron microscopy (EM; Rustom et al., 2004; Lokar et al., 2010). However, two major hurdles in this context are the impossibility to observe live cells and the difficulties to preserve both the fragile nanotubular structure and lipid distribution in the membrane (Rustom et al., 2004; Bell and Oparka, 2011; Sonnino and Prinetti, 2013). Indeed, EM requires multiple preparation steps susceptible to alter cellular structures and to generate artifacts. Development of fixation procedures allowing a better preservation of the structures, such as high pressure freezing, may overcome some of these issues (Vanhecke et al., 2008). EM can be coupled with fluorescence imaging studies, thus enabling to combine contextual information obtained by fluorescence microscopy in live cells with the resolution of EM but also to study dynamic processes or rare events and/or structures (McDonald, 2009). Such correlative approaches offer very interesting perspectives in the study of TNT and PD formation and transfer function and are the most promising to answer still unresolved questions on the structural/functional diversity of the various TNT-like structures described to date (cf. Introduction, Abounit and Zurzolo, 2012).

Finally, because separating TNTs from the cell constitutes a technical challenge that may be difficult to overcome, an alternative non-targeted approach to resolve lipid distribution in intercellular conduits may come from the developments of imaging mass spectrometry techniques (IMS; Ellis et al., 2013). Although the current technical limitations of IMS are critical for the study of very thin and fragile structures like TNTs, research towards improvement in achievable resolution/sensitivity and in sample preparation procedures is very active, quickly expanding the possibilities of these techniques (Passarelli and Winograd, 2011; Passarelli and Ewing, 2013).

## PERSPECTIVES

Despite differences in their formation and architecture, TNTs and PDs present striking functional and structural similarities, which need to be thoroughly explored. The questions raised by their unusual nanotubular membrane conformation are largely overlapping; therefore data obtained**on TNT can be informative on PD membrane and vice versa. In these review we have underlined the importance of studying the lipid composition and dynamics in these structures, as they are likely to be key elements regulating their structure and function. In addition to the imaging techniques described above, biophysical approaches and computational models may also greatly contribute to extend our knowledge on this important subject. As it is often the case for emerging fields, we believe that the key for a better understanding of those fascinating intercellular communication highways lies in pushing the current technology to new applications and in the development of transkingdom and interdisciplinary studies.

## Conflict of Interest Statement

The authors declare that the research was conducted in the absence of any commercial or financial relationships that could be construed as a potential conflict of interest.
